# We can and do make a difference by improving medical radiation services in rural and remote locations

**DOI:** 10.1002/jmrs.244

**Published:** 2017-12-04

**Authors:** Tony Smith

**Affiliations:** ^1^ Department of Rural Health University of Newcastle Taree New South Wales Australia

## Abstract

This is an editorial referring to the benefits that medical radiation practitioners can provide to the communities they live and work in.

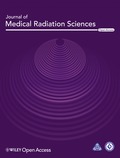

It is thought provoking to read articles in this issue of the *Journal of Medical Radiation Sciences (JMRS)* that reflect the important role that medical radiation professionals play in improving health service access and quality of care for the one‐third of the Australian population who live outside of the major cities. Many will be aware that the prevailing model of rural health has been the ‘deficit model’, which relates to the inability of regional, rural, and remote residents to access health care services that are relatively much more available in the cities.^1^ Of course, the outcome of poor service access is poor health outcomes. Much has been published and reported over many years about the poorer health outcomes of rural populations, as ways are explored to address the health disparity between rural and metropolitan populations. The articles in this issue of *JMRS* encourage readers to pause and reflect on how medical radiation health professionals can and do provide genuine benefits to regional, rural, and remote patients and communities.

It has been known for some time that, while the incidence of new cancer cases diagnosed is lower in remote locations than in more populous areas, the reverse is true of cancer deaths. For example, according to the Australian Institute of Health and Welfare (AIHW), between 2008 and 2012, the age‐standardised incidence for all cancers combined was 10.5% higher in ‘inner regional’ compared to ‘very remote’ areas; however, in ‘very remote’ areas cancer mortality between 2010 and 2014 was 13.8% higher than in ‘major cities’.^2^ Clearly, one of the contributing risk factors to the overall cancer death rate is access to cancer treatment. To help address that issue, the Australian Government funded a number of Regional Integrated Cancer Centres in regional population centres, which include radiation therapy facilities, to serve nonmetropolitan people. Is the potential of these Centres being met?

One of the articles compares radiation therapy utilisation (RTU) as a proportion of new cancer cases for different localities within the catchment of one of the Regional Cancer Centres.^3^ Data showed higher RTU rates after compared to before the installation of the radiation therapy unit. This is encouraging, as it suggests that cancer patients who may have otherwise not opted for radiation therapy treatment when they had to travel to Sydney or Newcastle, have, since commissioning of the Centre, accessed radiation therapy closer to home. This has major advantages for them, such as less time away from family and friends and considerably less financial burden.^4^ However, the authors of the article acknowledge the ongoing concern that for new cancer patients from remote locations RTU rates apparently decreased after compared to before the regional cancer care centre was opened. That finding is interesting in itself, as well as perhaps suggesting a need for further research. Nevertheless, for medical radiation professionals, other than the opportunity for further research, we can immediately gather from the article that the establishment of a radiation therapy service, with the employment of rural radiation therapists, has undoubtedly made a difference to the lives of those cancer patients who have been able to access treatment locally.

As well as access, the quality of care is also a priority. In another of the articles, the authors used standard outcome measures to assess the efficacy and toxicity of radiation therapy cancer treatment at a Regional Cancer Centre following radical prostatectomy.^5^ The retrospective, quality assurance audit of patient records showed that, using state‐of‐the‐art, evidence‐based radiation oncology practice, outcomes met national and international benchmarks. The authors concluded that there is ‘no detriment’ to those patients treated at the Regional Cancer Centre. This is important, as the quality of care for patients should be no different whether they are treated in a regional or a metropolitan cancer care centre.

Another relevant article is about the use of videoconferencing for remote supervision and support of limited licence X‐ray operators in rural and remote Queensland.^6^ The licensing of non‐radiographer X‐ray operators has been a controversial issue in diagnostic imaging for decades. It is only in more recent times that the profession has come to terms with the fact that, because Australia is such a large landmass with a small, dispersed population (except for a few more densely populated areas along the coastal fringe), some rural and remote Australians are potentially deprived of even basic general radiography services. Of course, the argument is that people who are isolated from such main‐stream diagnostic services should be prepared to travel to access them, and they often do. That argument, however, is fragile under some circumstances and fails to take into account the costs (in terms of both money and time) and the disadvantage of having to travel long distances to have, for example, a wrist X‐ray that then turns out to be normal.

The counter‐argument is that in locations where the X‐ray examinations are required too infrequently to justify employing a radiographer (as few as one or two a week^7^), a limited range of general radiography examinations could be performed locally by a non‐radiographer who has been specifically trained for that task and subsequently licensed under the relevant radiation control legislation. Ideally, of course, they would be supervised and supported by a radiographer. So it is that limited licence X‐ray operator radiography is legislated in each Australian State, even though, in fact, the licensing conditions, range of examination types and continuing education, monitoring, and support requirements differ from State to State. Indeed, that inter‐State variability is a cause for concern, which should be further investigated with a view to a common national policy, protocols, and procedures.

For most rural radiographers, therefore, the existence of limited licence X‐ray operators is no longer a point of argument, as it was in the 1980s. However, the existence of limited licensees remains a challenge for the medical radiation profession. The challenge is that rural and remote residents who have a plain general radiography examination performed by a non‐radiographer X‐ray operator should be assured that they will receive a service that is safe, of reliable diagnostic accuracy, and results in radiation exposure that is as low as reasonably achievable. That is, the quality of the examination should be comparable to the same examination having been performed by a radiographer. Hence, while some may disagree, there is a sound argument that the medical radiation profession as a whole, and local rural radiographers in particular, should take at least some degree of responsibility for the quality of services provided by non‐radiographer limited licence X‐ray operators. It is up to us, as a profession, to provide the necessary education, training, monitoring, and support to the X‐ray operators and, thus, the assurance for rural and remote populations that X‐ray operator radiography is of reasonable quality to meet patients’ health care needs.

The use of videoconferencing in combination with tele‐radiography shows promise as a means of both providing education and supervision to limited licence X‐ray operators, as well as in assessing the quality of the images they produced and providing them with constructive feedback.^6^ It was evident from the results of the study reported in *JMRS* that the images produced with videoconferencing supervision scored better on all quality criteria than images produced without, demonstrating a clear benefit when radiographers are involved, even if remotely. This highlights the fact that, even at distance from the location where the examination is being performed, the involvement of a diagnostic radiographer in general radiography examinations performed by non‐radiographer X‐ray operators can make a difference, improving both the service and image quality and, thus, potentially improving the health care outcomes for patients.

Those of us who work in the field of rural health workforce capacity building have become increasingly aware of late of the negative impression created by spruiking the deficit model of rural health. Some young, potential future practitioners may develop the idea that living in a rural or remote location is itself deleterious to your health and that working as a rural health professional is fraught with seemingly insurmountable challenges. In reality, the opposite is true. Not only does living in a regional, rural, or remote location offer significant lifestyle benefits, but being a health professional in a rural community can be, and usually is, extremely rewarding. In terms of lifestyle, there are environmental benefits on your doorstep, house prices are more affordable, and you only travel a few minutes to work and home again each day, and so have more leisure time. On the professional front, you can make a genuine and perceptible difference to peoples’ health and well‐being and you are overtly and often lauded as a highly valued member of the community.

Being consumed by the ‘daily grind’, it is easy to forget the difference that we, as health professionals, make to peoples’ lives. We can and do make a difference and the articles referred to above testify to that reality in the context of rural practice. ‘Making a difference’ by helping to bridge the health differential between rural and metropolitan populations is commonly recognised as a specific characteristic of rural practice. However, it is not uniquely rural. All medical radiation professionals, no matter their geographical location, have the capabilities to make a difference to the health and well‐being of the communities in which they live. It is worth reminding ourselves of this at times, and of the importance of recording and publishing the positive health outcomes that are brought about because of our professional knowledge, skills, and abilities.
